# Lip repositioning technique. A simple surgical procedure to improve the smile harmony

**DOI:** 10.4317/jced.54721

**Published:** 2018-04-01

**Authors:** Vicente Faus-Matoses, Ignacio Faus-Matoses, Ana Jorques-Zafrilla, Vicente J. Faus-Llácer

**Affiliations:** 1 DDS, MSc, PhD. Co-director of the Master of Restorative Dentistry and Endodontics, Department of Stomatology, Medicine and Dental School, Valencia University, Spain; 2 DDS, MSc, PhD. Professor of the Master in Orthodontics, Department of Stomatology, Medicine and Dental School, Valencia University, Spain; 3 Postgraduate Student, Valencia University, Spain; 4 MD, DDS, PhD. Director of the Master of Restorative Dentistry and Endodontics, Department of Stomatology, Medicine and Dental School, Valencia University, Spain

## Abstract

Excessive gingival display is an esthetic concern for patients. It is a condition in which an overexposure of the maxillary gingiva (>3mm) is present during smiling. There are different etiologies of a gummy smile, such as vertical maxillary excess, short and hyperactive upper lip, altered passive eruption, anterior dentoalveolar extrusion, or a combination of these causes. The correct diagnosis of all etiologic factors is imperative for its appropriate management. Many techniques have been used to restore the dentogingival relation for the management of gummy smile. Lip repositioning is a conservative surgical technique used to treat excess gingival display. It is a largely unknown treatment modality. This limits lip elevation on smiling and increases lip fullness. This technique was designed to be shorter, less aggressive and to have fewer postoperative complications compared to orthognathic surgery. In the current case series presents three patients who were successfully managed with lip repositioning. The aim of this article is to describe the lip repositioning technique to decrease gummy smile by a simple surgical procedure.

** Key words:**Lip repositioning, gummy smile, smile harmony.

## Introduction

Excessive gingival display (EGD), commonly termed gummy smile, is a condition characterized by an overexposure of the maxillary gingiva while smiling (1). It is distinguished by showing more than 1.5-2 mm of the gingiva (2). The amount of discrepancy considered unattractive varies between populations; however, an excess of more than 3 mm is agreed upon worldwide (1-3).

EGD may result from a single discrepancy, but is more commonly the result of an interplay of multiple factors. Proper diagnosis of etiological factors is essential to select the right treatment protocol. The aetiology of EGD is variable: related to bony maxillary excess, related to conditions causing gingival enlargement, related to deficient maxillary lip length or related to excessive mobility of maxillary lip (4).

Many techniques have been used to restore the dentogingival relation for the management of gummy smile. Such techniques include crown lengthening procedures, orthodontic leveling of the gingival margins of maxillary teeth, maxillary teeth intrusion, lip repositioning, orthognatic surgery and nonsurgical procedures like the use of the botulinum toxin (5). Anterior dentoalveolar extrusion is treated with orthodontic intrusion and vertical maxillary excess is treated with orthognatic surgery. However, in cases with minor vertical discrepancy, the cost, invasiveness and postoperative morbidity of the procedure cannot always be justified for the outcome achieved (1).

Lip repositioning procedure was first described in 1973 by Rubinstein and Kostianovsky as part of medical plastic surgery. Later on, it was introduced in dentistry, after being modified in 2006 by Rosenblatt and Simon. It is a conservative permanent surgical technique that offers a less invasive approach to EGD. The surgery aims to limit smile muscle pull (zygomaticus minor, levator anguli, orbicularis oris, and levator labii superioris) by reducing the depth of the upper vestibule (2).

The aim of this article is to describe the lip repositioning technique to decrease gummy smile by a simple surgical procedure.

## Case Reports

-Patient profiles and consent

**Figure 1:**
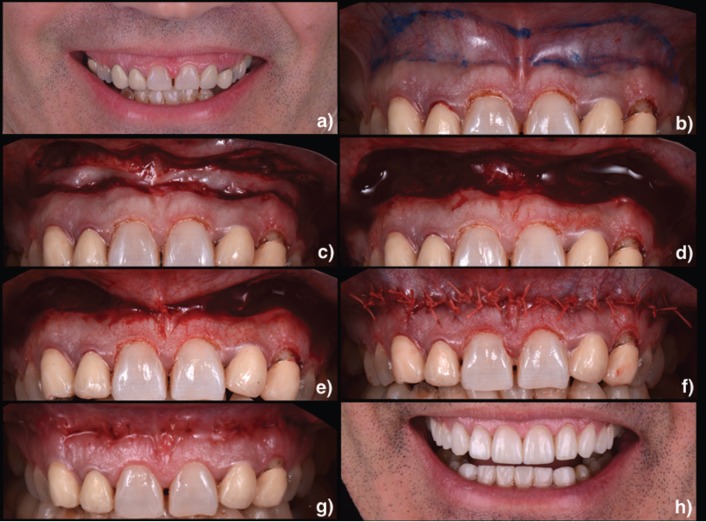
a) Preoperative image of the dynamic smile. b) A marking pencil was used to identify the apical, coronal and lateral boundaries of the incision. c) Incision. d) Exposed connective tissue after the epithelial excision. e) Suturing was first initiated at the midline using interrupted nonresorbable suture. f) The mucosa was advanced and sutured to the attached gingiva at the mucogingival junction using multiple interrupted sutures. g) Situation after the suture removal. h) Final situation.

**Figure 2:**
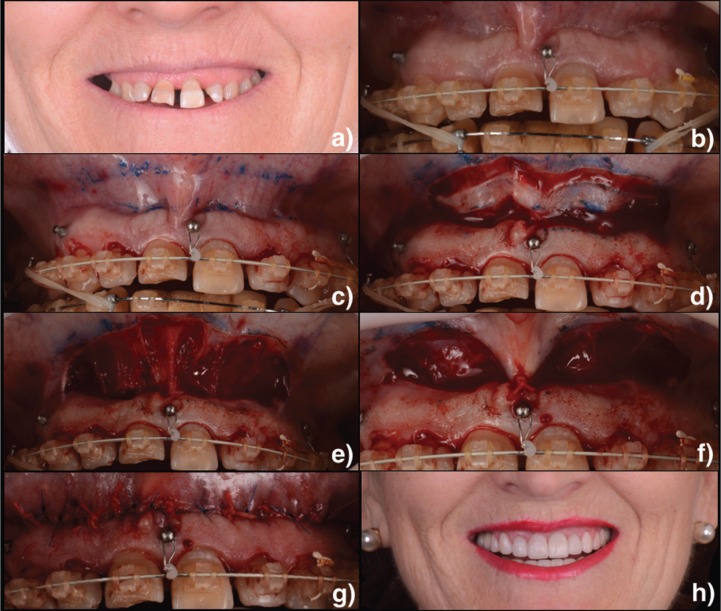
a) Preoperative image of the dynamic smile. b) Intraoral preoperative image. c) Borders of the surgical excision were marked. d) Incision. e) Exposed connective tissue after the epithelial excision. f) Suturing was first initiated at the midline using interrupted nonresorbable suture. g) The mucosa was advanced and sutured to the attached gingiva at the mucogingival junction using multiple interrupted sutures. h) Final situation after surgical and restorative treatment.

Three patiens, aged from 48 to 65 years, presented between 2014 and 2016 with the chief complaint of a “gummy smile” (Fig.1a, 2a, 3a). They had previously been treated with orthodontics (Fig. 2b) and esthetic crown lengthening. All of them were categorized as having EGD. Written informed consent was obtained following a discussion of risks, benefits, and treatment alternatives. Intra– and extraoral photographs were taken for planning and records.

**Figure 3:**
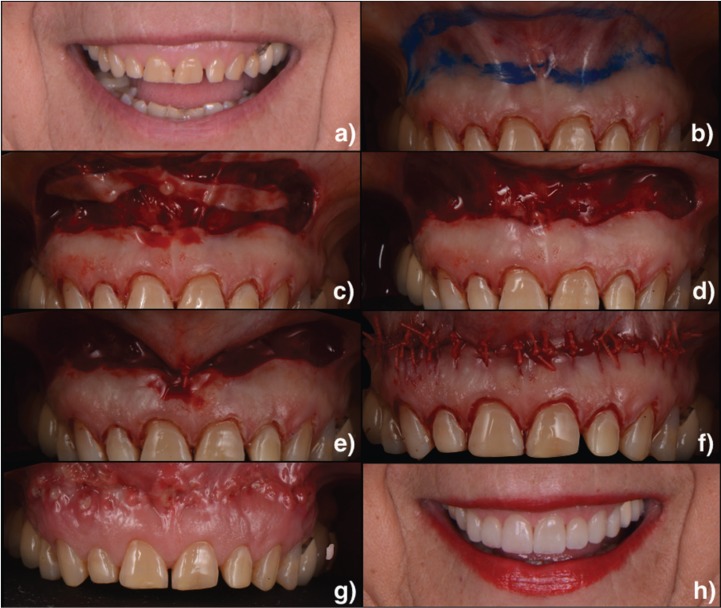
a) Preoperative image of the dynamic smile. b) Borders of the surgical excision were marked. c) Incision. d) Exposed connective tissue after the epithelial excision. e) Suturing was first initiated at the midline using interrupted nonresorbable suture. f) The mucosa was advanced and sutured to the attached gingiva at the mucogingival junction using multiple interrupted sutures. g) Situation after the suture removal. h) Final situation.

-Surgical procedure

First, adequate local anesthesia was achieved. The technique consists in doing an elliptical incision in the depth of the vestibule. A marking pencil was used to outline the borders of the elliptical incision (Fig.1b, 2c, 3b). The inferior border of the incision was placed at the mucogingival junction and was extended from the mesial aspect of the first premolars bilaterally. As a general rule, it has been suggested that the distance between the superior and inferior borders must be twice the length of repositioning desired in the smile. Partial-thickness incisions were made using a scalpel across the superior and then the inferior border (Fig.1c, 2d, 3c). The outlined mucosa is removed by partial thickness dissection, exposing the underlying connective tissue (Fig.1d, 2e, 3d). The area of frenectomy was approximated with a simple interrupted suture to ensure symmetry and proper midline placement (Fig. 1e, 2f, 3e). The remaining closure bilaterally was completed with interrupted sutures to stabilize the new mucosal margin to the gingiva. Nonresorbable sutures were used (3-0 silk) (Fig.1f, 2g, 3f).

-Postoperative instructions 

Prescriptions for analgesics (ibuprofen 600 mg every 8 hours as needed) and chlorhexidine gluconate 0.12% (gentle bathing of the surgical area twice daily for 2 weeks) were given. Patient was instructed to apply ice packs at 20 minute intervals for 24 hours and soft diet during the first postoperative week. Oral hygiene can be reinstated after 48 hours. Additional instructions include avoiding any manipulation or mechanical trauma to the surgery and minimizing lip movements when smiling or talking the first 2 weeks postoperatively. Sutures were removed at the 1-week postoperative visit (Fig. 1g, 3g). The extraoral final situation can be observed in Figures 1h, 2h and 3h.

## Discussion

The aim of this article is to describe the lip repositioning technique to decrease gummy smile by a simple surgical procedure. This technique was designed to be shorter, less aggressive and to have fewer postoperative complications compared to orthognathic surgery (7).

Proper diagnosis of the etiological factors is the first step to select the right treatment protocol. The aetiology of EGD is variable. It may include extraoral or introaoral components. The contraindications for this technique include the presence of a minimal zone of attached gingiva, which can create difficulties in flap design, stabilization and suturing. Another contraindication is several vertical maxillary excess (VME). Degree II VME has gingival and mucosal display of 4 to 8 mm. In the other hand, in degree III VME more than 8 mm of soft tissue are seen. In both cases, an interdisciplinary approach is required (7).

In order to determine other factors that are not related with the hyperfunction of the upper lip elevator muscle, certain characteristics must be taken in account. Facial proportions must be symmetric in the three horizontal thirds, without identification of higher proportion of the inferior third, which could characterize an excessive maxillary vertical growth. Another factor to be evaluated is the distance between the gingival margin and CEJ, which ideally is < 1.5 mm. Distances greater than 1.5 mm indicate an excessive gingival tissue covering the tooth crown, typical in altered passive eruption. Finally, the crown length-height relation must be evaluated. The maxillary central incisor’s width must be about 80% of its length, with an accepted variation between 65% and 85%, and the maxillary lateral incisors about 70% (3).

The technique described in the present cases is a modification of the original technique by Rubinstein and Kostianovsky, initially used in medical plastic surgery and adapted for use in dentistry (1-6). There is a modified technique in which the maxillary labial frenulum is maintained and two mucosal strips, one at each side of the frenulum, are removed (3). Leaving the frenulum intact helps maintaining the position of the labial midline, prevents changes in lip symmetry and decreases the morbidity associated with the procedure, but limits the possibility of correcting EGD in the region of the maxillary central incisors (1). For this reason, in the present cases not to maintain the frenulum was decided.

Some authors believe that using a reversible procedure prior to definitive surgery is currently the best way for both the patient and doctor to preview the intended result before moving forward with elective surgery. It consists in doing a mark along the proposed surgical resection. Once the area is marked, sutures are used to complete the reversible procedure. This allows the upper board to be drawn down to the mucogingival junction, inverting and tucking behind the tissue proposed for excision. At this point, photographs are taken and the patient is able to evaluate the potential result (6).

Despite, the limited availability of the studies focused on the outcome of lip repositioning, the systematic review published by Tawfik *et al.* showed that lip repositioning successfully improved EGD by 3.4 mm (2). The current study indicates that after 1-year follow-up, this technique can produce stable results.

In conclusion, lip repositioning technique is a simple procedure that offers an excellent alternative to other procedures with higher morbidity rates. In the present cases, the functional and aesthetic parameters required by the patients were achieved and they were satisfied with the outcome of the procedure.
